# Challenges and barriers to HIV service uptake and delivery along the HIV care cascade in Cameroon

**DOI:** 10.11604/pamj.2020.36.37.19046

**Published:** 2020-05-27

**Authors:** Albert Frank Zeh Meka, Serge Clotaire Billong, Ismael Diallo, Ousseni Wendlassida Tiemtore, Brian Bongwong, Georges Nguefack-Tsague

**Affiliations:** 1 National Aids Control Committee, Ministry of Public Health, Yaounde, Cameroon; 2 Faculty of Medicine and Biomedical Sciences, University of Yaounde, Yaounde, Cameroon; 3 Centre Hospitalier Universitaire Yalgado Ouedraogo, Ouagadougou, Burkina-Faso; 4 Initiatives Conseil International-Santé (ICI-Santé), Ouagadougou, Burkina-Faso

**Keywords:** Cascade, stock-outs, barriers

## Abstract

**Introduction:**

The year 2017 marked a transition period with the end of the implementation of Cameroon´s 2014-2017 HIV/AIDS National Strategic Plan (NSP) and the development of the 2018-2022 NSP. We assessed barriers and challenges to service delivery and uptake along the HIV care cascade in Cameroon to inform decision making within the framework of the new NSP, to achieve the UNAIDS 90-90-90 target.

**Methods:**

We conducted a cross sectional descriptive study nationwide, enrolling HIV infected patients and staff. Data were collected on sociodemographic characteristics, HIV testing, antiretroviral therapy and viral load testing delivery and uptake and factors that limit their access.

**Results:**

A total of 137 staff and 642 people living with HIV (PLHIV) were interviewed. Of 642 PLHIV with known status, 339 (53%) repeated their HIV test at least once, with range: 1-10 and median: 2 (IQR: 1-3). Having attained secondary level of education (OR: 2.07, 95% CI: 1.04-4.14; P=0.04) or more (OR: 2.91, 95% CI: 1.16-7.28; P=0.02) were significantly associated with repeat testing. Psychological (refusal of service uptake and existence of HIV), community-level (stigmatization and fear of confidentiality breach) and commodity stock-outs “HIV test kits (21%), antiretrovirals (ARVs) (71.4%), viral load testing reagents (100%)” are the major barriers to service delivery and uptake along the cascade.

**Conclusion:**

We identified individual, community-level, socio-economic and health care system related barriers which constitute persistent bottlenecks in HIV service delivery and uptake and a high rate of repeat testing by PLHIV with known status. Addressing all these accordingly can help the country achieve the UNAIDS 90-90-90 target.

## Introduction

The continuum of care (also referred to as the cascade of care) for successful HIV treatment includes: HIV testing, linkage, engagement in care, and retention on antiretroviral therapy (ART) with viral suppression as the ultimate clinical goal to improve individual health outcomes of people living with HIV/AIDS (PLHIV) and reduce HIV acquisition and transmission, thus conferring community and public health benefits [1]. Over the last decade, millions of individuals in sub-Saharan Africa (SSA) have started ART, however, low HIV testing rates and losses between the point of testing and the initiation of ART have mitigated this success, and the majority of people in need of treatment are not receiving it [2]. Several studies have described the performance of service delivery and uptake or dwelled on barriers to uptake of services and attrition along the HIV care continuum cascade for the general population and in pregnant women across the world including SSA [3-11]. The factors identified are individual, socio-economic or community-level or health system related factors. Efforts are needed to optimize the HIV care continuum in order to achieve the 90-90-90 target, that is by 2020, 90% of all people living with HIV will know their HIV status, 90% of all people with diagnosed HIV infection will receive sustained antiretroviral therapy and 90% of all people receiving antiretroviral therapy will have viral suppression [12]. Key to these efforts will be to overcome and/or eliminate legal, social, environmental, and structural barriers that prevent PLHIV from accessing and utilizing HIV services [13]. In 2015, Cameroon subscribed to the UNAIDS 90-90-90 ambitious target which aims to end the AIDS epidemic in 2030. Several efforts have therefore been undertaken by the Cameroonian government to provide HIV services to all Cameroonians by breaking financial, social and geographical barriers in order to achieve this target in 2020. As of 31^st^ December 2016, 2 418 986 HIV tests were done but the number of people tested is not known. Also, the ART coverage amongst PLHIV was 32% [14] far below the 60% objective set by the 2014-2017 NSP. Furthermore, only 46 993 viral load tests were done within the same year amongst the 168 349 PLHIV on ART at the end of the year 2015. As we get close to the year 2020, to achieve the 90-90-90 ambitious target, ART has experienced a rapid scale-up with the adoption of the test and treat strategy, systematic offer of HIV-testing in health facilities, decentralisation through the creation of more ART and option B+ sites across the country, community dispensation of ART to stable PLHIV on ART and the enrollment of 8 reference laboratories to conduct viral load (VL) testing covering the 10 regions of the country and reduction of the cost of the test. It is in this context that we sought to explore factors associated with attrition along the HIV care cascade in an attempt to contribute in the reorientation and reinforcement of strategies within the framework of the new 2018-2022 HIV/AIDS NSP, so as to achieve the UNAIDS ambitious 90-90-90 target. More specifically, we sought to determine the magnitude of repeat testing among PLHIV with a previous HIV diagnosis.

## Methods

**Study setting**: the study was conducted on the national scale in health facilities (ART and PMTCT services, laboratories, and outpatient department), regional drug procurement/distribution institutions (regional funds for the promotion of health (RFPH)), regional technical groups (RTG) for the fight against AIDS and reference laboratories for viral load testing (a total of 8 laboratories were operating as of 31 December 2016). ART and PMTCT sites provide HIV screening and treatment services to the general population and pregnant women respectively. There are two categories of ART sites: approved treatment centers (ATC) which are found in health facilities of 1^st^, 2^nd^ and 3^rd^, categories of our health system and HIV management units (HMU) found in 4^th^, 5^th^ and 6^th^ category facilities.

**Study design and population**: we conducted a cross-sectional and descriptive study from 8^th^ May to the 10^th^ of July 2017 to assess service delivery and uptake at the various stages of the HIV care cascade. Participants for this study were PLHIV enrolled on ART and staff in: i) health facilities (ART services, PMTCT services, laboratories and outpatient department): medical doctors and nurses, ii) national and regional drug procurement and distribution institutions (pharmacist or ware house in-charge) and iii) HIV viral load testing reference laboratories (laboratory in-charge), iv) mobile HIV testing units in RTGs (psychosocial support agents).

**Sampling and data collection**: we sampled sites distributed around the 8 national viral load reference laboratories, a total of 193 facilities were involved. The list of facilities distributed around the country with the numbers of regular PLHIV ART users per site as of 31^st^ December 2016 were obtained from the National Aids Control Committee (NACC). The selection of health facilities was then done by stratified sampling with probability proportional to the size of regular ART users per site. As variables for stratification we used the regions (all 10 regions of the country were involved), the location (urban/rural), type of ART site in the facility (ACT/HMU) and ownership (private/public). A total of 25 sites were sampled nationwide of which 12 rural and 13 urban, 9 ATC and 16 HMU, 19 public and 6 private. The sample size of PLHIV was calculated using the formula:

$$n=\frac{Z_{1}-\alpha /\overset{2}{2}P\left(1-P\right)}{d^{2}}$$

[15]

Where n = sample size, Z_(1-α/2 )_= upper (1-α/2) quantile of the standard normal distribution, P= proportion of PLHIV with a previous HIV diagnosis who repeat their HIV test (we don´t have much information on the subject to begin with, so we assumed that half of PLHIV repeat their HIV test after previous HIV diagnosis) and d= precision. Assuming a level of precision of 4% and a confidence level at 95%, we calculated a sample size n= 1.962 x 0.5 x (1-0.5)/0.042 = 600 PLHIV. An additional 10% was added to account for non-respondents, resulting in a sample size of n= (0.1x600) + 600 = 660. The sample size obtained was then distributed to the sites proportionally to the size of regular PLHIV ART users. A systematic sampling technique was used to select PLHIV in ART sites from a list which was drawn and numbered. Staff were conveniently sampled. Data was collected with the use of semi-structured questionnaires which were administered in the study sites during a face-to-face interview by trained interviewers in the participants´ preferred official language (French or English). Interviewers were made up of staff from NACC, RTG and facilities (psychosocial agents). The questionnaires included sociodemographic characteristics, knowledge and questions regarding HIV testing, care and treatment and viral load testing service delivery and uptake.

**Statistical analysis**: at the end of data collection, questionnaires were sorted out to check for errors and any missing information. Data was entered and analysed with Epi info 7 software. Data double checking was done before analysis to ensure consistency and accuracy. Some variables were recoded to facilitate analysis. We used descriptive statistics in the form of proportions (%) for categorical data, mean if data followed normal distribution with standard deviation otherwise median with interquartile range for continuous data. Chi-square and Fisher´s exact test (if expected counts less than 5) were used to compare proportions and associations with outcome variables (repeat testing and ART discontinuation). For bivariate analyses, associations between outcome variable and covariates were quantified using odd ratios (OR) with 95% confidence interval (CI). Variables found to be associated with p-values less than 0.25 were reexamined in a logistic regression model. P-values less than 0.05 were considered statistically significant.

**Ethical considerations**: administrative authorizations (hospital, regional fund and viral load testing laboratory directors, regional coordinators for the fight against HIV) were obtained. Ethical clearance was obtained from the national ethics committee for human research. Informed consent was also obtained from study participants and each was assigned a unique identification code which was written on the questionnaire.

## Results

A total of 779 participants were interviewed, of which 137 staff and 642 PLHIV. Amongst PLHIV (N=642), the 30-39 (30%) and 40-49 years (35%) age groups and women (68%) were most represented. The age ranged from 12 to 78 years and the mean age was 41±11.03 years. Most PLHIV interviewed were married (43%), and more than half attended secondary school while more than 1/4 had never been to school (Table 1). As shown in Table 2 we assessed repeat of HIV testing among PLHIV with a previous HIV diagnosis within the first 3 months of HIV diagnosis and the lifetime HIV positive testing. More than half of PLHIV repeated their HIV test since they were known HIV positive (53%, 339/642) while 45% (287/642) repeated their test within the first 3 months of HIV diagnosis. Overall, most retesters repeated HIV tests 1-3 times (Range 1-10), with a median of 2 (IQR:1-2) tests within the first 3 months of HIV diagnosis. Patients with secondary school (OR: 2.07, 95% CI: 1.04-4.14; P=0.04) and higher level of education (OR: 2.91, 95% CI 1.16-7.28; P=0.02) were significantly more likely to be repeat testers compared to those with no education.

**Table 1:** Socio-demographic characteristics of patients

Variables*	Patients	
Age (years)	N	Percentage (%)
10-19	8	1
20-29	84	13
30-39	191	30
40-49	225	35
50-59	97	15
≥60	32	5
Sex		
Male	204	32%
Female	436	68%
Marital status		
Single	219	34.4
Divorced	27	4.2
Married	274	43
Separated	18	2.8
Widow/widower	99	15.5
Level of education		
None	168	29
Primary	330	57
Secondary	39	7
Higher	45	8

* Missing values

**Table 2:** Assessment of repeat testing among PLHIV with known status (n=642)

Assessment of repeat HIV testing among PLHIV with previous HIV diagnosis (n=642)				
Variables	Yes		No	
	N	%	N	%
Repeated test within 3 months following 1st positive test	287	45	355	55
Ever repeated HIV test since known HIV+ (lifetime positive testing)	339	55	303	47
**Number of HIV tests done**	**Number of positive HIV testing within 3 months following initial HIV+ test (n=274)**		**Number of lifetime positive HIV testing (n=339)**	
	**N**	%	**N**	%
1	123	44.9	124	36.6
2	81	29.6	108	31.9
3	44	16.1	56	16.5
4	11	4.0	22	6.5
5	8	2.9	11	3.2
≥6	7	2.5	18	5.3
**Multivariate assessment of factors associated with repeat HIV testing among PLHIV with previous HIV diagnosis (n=393)**				
**Variable**	**Value**	**Odds ratio (95% CI)**	**P-value**	
**Sex**	Male (vs Female)	0.73 (0.49-1.07)	0.11	
**Marital status**	Divorced (vs single)	1.17 (0.48-2.81)	0.73	
	Married (vs single)	1.0 (0.67-1.47)	0.97	
	Separated (vs single)	1.50 (0.57-4.09)	0.40	
	Widow/widower (vs single)	0.69 (0.41-1.16)	0.16	
**Level of education**	Primary (vs uneducated)	1.28 (0.62-2.63)	0.50	
	Secondary (vs uneducated)	2.07 (1.04-4.14)	0.04	
	Higher (vs uneducated)	2.91 (1.16-7.28)	0.02	

Barriers to HIV testing were identified at all levels: health system, community-level and patient-level. PLHIV (N=995) reported stigmatisation (53%), fear of confidentiality breach (21%) and insufficient counselling (11%) as major barriers to HIV screening uptake while staff (N=146) reported refusal by patients to get tested (25%), HIV test kits stock-outs (21%), and fear to know HIV status (10%) as the major barriers to HIV screening delivery. Also, 5% (52/995) of PLHIV and 8% (11/146) of staff in health facilities reported the cost of the HIV test was a barrier to HIV testing (Table 3). On assessment of barriers to ART uptake by PLHIV (n=986), stigmatization (51%), fear of confidentiality breach (20%) and insufficient counseling (12%) were the most reported while staff reported refusal by patients to initiate ART (2/5) and their clinical condition (2/5) as the most common barriers to ART delivery (Table 4). In health facilities and regional drug procurement and distribution institutions, most staff interviewed (20/28, 71.4%) reported pediatric ARV stock-outs within the last 3 months before the study and the length of stock-outs ranged from 7 to 60 days (Table 5).

**Table 3:** Barriers to HIV testing uptake and delivery

BARRIER	Reported by PLHIV		Reported by staff	
	N	%	N	%
Stigmatization	532	**53**	8	5
Fear of confidentiality breach	211	**21**	20	**14**
Insufficient counselling	111	**11**	17	**12**
Cost*	52	5		
Distrust in health system	26	2.6		
Don´t know	23	2.3		
Apparent good health	14	1.4		
Death of husband	6	0.6		
Denial of HIV existence	5	0.5		
Distance to the health facility	4	0.4		
Fear to know HIV status	3	0.3		
Shame	2	0.2		
Ignorance	2	0.2		
Refusal to get tested	1	0.1		
Others**	3	0.3		
HIV tests stock-outs			30	**21**
Heavy workload			8	5

* cost of HIV test as per government recommendation: 0.87 USD

** Lack of courage, negligence, too much self confidence

**Table 4:** Barriers to ART uptake and delivery

Barriers	N	Percentage (%)
**Reported by staff (n=5)**		
Patient's clinical condition	2	40
Spiritual and herbal therapies	1	20
Refusal to initiate ART	2	40
**Reported by PLHIV (n=986)**		
Stigmatization	498	51
Fear of confidentiality breach	199	20
Insufficient counselling	114	12
Indirect cost of ART initiation (transport, consultation fees, patient record, work-up)	91	9
Fear of side effects	19	1.9
No reason	16	1.6
Advice received	7	0.7
Denial of HIV existence	7	0.7
Distance from ART site	6	0.6
Fear to engage in life treatment	6	0.6
Shame	5	0.5
Ignorance	4	0.4
Long waiting time to be attended by health care provider	4	0.4
Refusal to accept HIV status	3	0.3
Others*	7	0.7

* Negligence, don´t know, lack of courage, herbal and spiritual therapies, distrust in health system

**Table 5:** History of ARV stock-outs in ART sites and supplying institutions

Variables	N	%
ARV stock-outs within the last 3 months* (n=28)		
Yes**	20	71.4
No	8	28.6
**Length of pediatric ARV stock-outs within the last 3 months (n=4)**		
7 days	1	25
14 days	1	25
30 days	1	25
60 days	1	25

* Staff from ART sites and Regional Funds for the Promotion of Health, which supply all health facilities with ARV

** Staff reported only pediatric ARV experienced stock-outs. The mean length of pediatric ARV stock-outs was 28 days

Concerning viral load testing delivery and uptake, of the 7 VL reference laboratories surveyed, 6 reported interruption of viral load testing delivery within the last 12 months before the study and all these laboratories encountered reagent stock-out as main reason for interruption. Other reasons included VL testing machine breakdown (1/6) and staff shortages (1/6) (Table 6). Despite the fact that VL testing was recently instituted as a work-up for follow-up, 71% (456/642) of patients interviewed were aware of VL testing and 78% of these (358/456) had performed a VL test at least once since they initiated ART. Viral load testing reagent stock-out (49%), viral load testing machine breakdown (12%), lack of information/ignorance (9%) and distance to laboratory (6%) were the most reported barriers to VL testing delivery (Table 7). We assessed staff availability for the delivery of services along the HIV care cascade, of 106 staff interviewed in facilities, 53% (56/106) reported staff shortage.

**Table 6:** History of and reasons for interruption of viral load testing activities in reference laboratories within the last 12 months before the study

Variable	N	%
**History of VL testing delivery interruption (n=7)**		
Yes	6	86
No	1	14
**Reasons for VL testing interruption (n=6)**		
**Reagent stock-out**		
Yes	6	100
**VL machine breakdown**		
Yes	1	17
No	5	83
**Limited staff**		
Yes	1	17
No	5	83

**Table 7:** Barriers to viral load testing

Barriers	N	%
VL reagent stock-out	55	49
VL machine breakdown	13	12
Lack of information/ignorance	10	9
Negligence	7	6
Distance to VL laboratory	7	6
Discouraged by long waiting time for results to be returned	4	4
Fear to know result	4	4
Cost of transport to VL testing lab	3	3
Don't know where it is done	3	3
Time constraints to go and do the test	3	3
Others*	3	3
**Total**	**112**	**100**

* Never prescribed, don´t know, distrust in test

## Discussion

In our study we found a high proportion of repeat HIV testing amongst PLHIV who know their status, especially within the first 3 months following initial diagnosis. Our reporting system double counts all the HIV positive individuals and sums them, thus inflating the number of PLHIV making it appear that there is a greater loss to follow-up between testing positive and enrolling in care than actually exists. This has a serious impact on planning and response to the HIV epidemic as resources might be wasted. Contrary to our findings, repeat/multiple testing has been mostly reported in people with prior HIV negative or unknown statuses [16-20]. Secondary and higher levels of education were associated with increasing odds of ever being tested, similar to findings in studies in SSA and Europe [16,17,20]. Sex was not associated with repeat HIV testing in our study as reported by Matković Puljić *et al*. in Croatia [16] and Hensen *et al.* in Zambia [17]. Forty per cent (40%) of people living with HIV are not aware of their HIV-positive status [21]. Refusal to get tested, HIV tests kits stock-outs, stigmatization and fear of confidentiality breach were the most reported barriers to HIV testing, consistent with reports and findings in systematic reviews and studies in countries in SSA [4,22-28].

This study reveals a wide spectrum of barriers to ART access reported by both patients and staff which undermine the ART programme. These factors are individual, community-level, socio-economic and health-care system related. Our findings are similar to those reported in other systematic reviews and studies in sub-Saharan Africa [5,8,10,28]. The benefits of virological monitoring for patients on ART are well established and include the ability to diagnose adherence problems and treatment failure, and optimize therapy to support reduced transmission [29]. Routine virological monitoring is rarely available in most high-HIV prevalence settings. Of the 7 reference viral load testing laboratories surveyed, 6 reported a break-off of viral load testing performance within 12 months before the study and all reported reagent stock-outs as the main factor. A wide range of factors, individual, socio-economic and health care system-related were reported in our study. In accordance with our results, a systematic review in low and middle income countries (LMIC) by Roberts *et al*. [30] revealed that a recent survey by WHO targeting 122 LMICs found that only 20% of ART patients receive VL testing. In the LMICs surveyed, there were only 2 VL instruments, on average, per 8 706 people on ART, with 10% of these machines not in operation because they had not yet been installed or required repair, or due to lack of reagents and the absence of staff training. Findings from an in-depth qualitative survey of experts based in India, Kenya, Malawi, South Africa, and Zimbabwe found that in Malawi, with >400 000 patients on ART, only 37 000 received a VL test in 1 year. Respondents cited financial constraints as a key reason for incomplete or slow implementation. In addition, insufficient and overburdened healthcare professionals, poor training and lack of knowledge, and weak transport and laboratory systems were all considered barriers to scale-up of VL testing. Most of the health facilities surveyed reported staff shortage in HIV health services delivery. The various steps in the cascade are heavy enough and need adequate staffing. Staffing problems in HIV service delivery have been reported in systematic reviews involving SSA [8,31].

## Conclusion

As Cameroon is scaling-up the fight against HIV by adhering to the UNAIDS 90-90-90 target with the objective to end the AIDS epidemic in 2030, there are still a lot of challenges and barriers to overcome to meet this target. Individual, community-level, socio-economic and health system and facility-related barriers constitute persistent bottlenecks in service delivery along the HIV care cascade which can undermine the achievement of the 90-90-90 target in 2020. Psychological factors (refusal of HIV existence, to get tested and to take ART), community-level factors (stigmatization and fear of confidentiality breach) and commodity stock-outs (HIV tests, ARV, viral load testing reagents) appear to be the major barriers to service delivery and uptake along the cascade. Repeat testing and staff shortage constitute major challenges to the reporting system and service delivery respectively. Findings from this study inform decision making within the framework of Cameroon´s 2018-2022 HIV/AIDS national strategic plan, the challenges and barriers identified should be taken into consideration in its implementation for better decision-making, and common efforts should be made by patients, staff and the decision-makers for successful achievement of the 90-90-90 target.

### What is known about this topic

- There exist barriers to HIV testing, access to antiretroviral therapy and viral load testing at the patient-level, community-level and health-system;
- Staffing shortage in the health system is a challenge for HIV service delivery.

### What this study adds

- There is a high rate of repeat testing among PLHIV with a previous HIV diagnosis which is not properly captured by the reporting system. Our data collecting system is still mainly paper-based, the use of unique identifier code, case based surveillance and electronic medical records therefore appear as necessities. There is also a need to understand and better address reasons for repeat testing among PLHIV with known status.

## Competing interests

The authors declare no competing interests.
